# Molecular Analysis of EBUS-TBNA Samples for Nodal Staging in Non-Small Cell Lung Cancer

**DOI:** 10.3390/cancers18111797

**Published:** 2026-06-01

**Authors:** Ahmed Ehab, Naoufal Benabdallah, Kaid Darwiche

**Affiliations:** 1 Pulmonary Medicine Department, DGD Lung Clinic Hemer, 58675 Hemer, Germany; naoufal.benabdallah@lkhemer.de (N.B.); kaid.darwiche@lkhemer.de (K.D.); 2 Pulmonary Medicine Department, Mansoura University, Mansoura 35516, Egypt

**Keywords:** EBUS-TBNA, NSCLC, molecular profiling, mediastinal staging, PD-L1, NGS

## Abstract

Since the discovery of EGFR mutations, treatment strategies have undergone major advances over the past two decades. Accordingly, molecular testing for oncogenic driver mutations is recommended at the time of diagnosis of advanced NSCLC. In addition, molecular analysis of lymph node samples has emerged as a promising tool for improving the accuracy of nodal staging in NSCLC. This approach may enhance the precision of staging in early-stage disease planned for curative surgical resection, thereby optimizing adjuvant treatment decisions and supporting complete resection at the time of surgery. In this review, we examine the importance and current status of molecular testing for oncogenic driver mutations in samples obtained via endobronchial ultrasound-guided transbronchial needle aspiration (EBUS-TBNA) or EBUS-guided cryobiopsy, and its role in improving the accuracy of nodal staging in NSCLC.

## Simple Summary

Non-small cell lung cancer remains the leading cause of cancer-related mortality worldwide and continues to be associated with poor outcomes despite advances in diagnosis and treatment. Recent progress in molecular diagnostics has transformed NSCLC management by enabling the identification of oncogenic driver mutations and resistance mechanisms, thereby facilitating the use of targeted therapies that improve outcomes in selected patients. Endobronchial ultrasound-guided transbronchial needle aspiration (EBUS-TBNA) has become an important minimally invasive procedure for both mediastinal staging and tissue acquisition for molecular testing. This review summarizes current evidence regarding the reliability of EBUS-TBNA samples for detecting key genetic and immune biomarkers relevant to targeted therapy and immunotherapy. In addition, factors influencing sample quality and emerging technologies, are discussed as potential tools to further enhance diagnostic accuracy and personalized treatment strategies.

## 1. Introduction

Non-small cell lung cancer (NSCLC), representing approximately 85% of lung cancer cases, remains the leading cause of cancer-related mortality worldwide and continues to be associated with poor outcomes despite advances in diagnosis and treatment [1]. Recent developments in comprehensive genomic profiling have significantly improved the molecular characterization of NSCLC. These technologies enable the identification of therapeutically actionable alterations in key oncogenic drivers, including epidermal growth factor receptor (EGFR), anaplastic lymphoma kinase (ALK), ROS proto-oncogene 1 (ROS1), and Kirsten rat sarcoma viral oncogene homolog (KRAS). The detection of such genomic alterations have facilitated the development and clinical implementation of targeted therapies, leading to substantial improvements in clinical outcomes for patients with metastatic disease [2].

Although surgical resection remains the standard treatment for early-stage and locally advanced NSCLC, postoperative recurrence rates remain high, reaching up to 70% within five years in some patient cohorts. This high recurrence rate underscores the need for improved risk stratification and more individualized treatment strategies [3]. Current clinical guidelines emphasize the importance of molecular diagnostics in NSCLC. The joint guideline issued by the College of American Pathologists (CAP), the International Association for the Study of Lung Cancer (IASLC), and the Association for Molecular Pathology (AMP) recommends routine molecular testing for oncogenic driver mutations at the time of diagnosis in patients with advanced NSCLC [4]. According to these recommendations, testing for *EGFR*, *ALK*, and *ROS1* alterations is mandatory, while additional genes should be included when sufficient tumor tissue is available [4,5]. Interestingly, emerging evidence indicates that actionable genomic alterations occur with comparable prevalence in both early-stage and advanced NSCLC. These findings support the implementation of comprehensive genomic profiling even in potentially curable disease stages, as such testing may provide clinically relevant information for treatment planning and prognostic assessment [6,7].

Beyond individual driver mutations, co-occurring genomic alterations also play an important role in determining tumor biology, prognosis, and therapeutic response. For example, mutations in *TP53*, which occur in approximately 50% of NSCLC cases, have been associated with poorer survival outcomes, including in tumors harboring *EGFR* mutations [8,9]. Similarly, alterations in genes such as Kelch-like ECH-associated protein 1 (KEAP1) and Serine/Threonine Kinase 11 (STK11) have been linked to resistance to immunotherapy and altered responses to immune checkpoint inhibitors [2].

## 2. Common Molecular Alterations in NSCLC

Molecular testing in NSCLC relies on complementary techniques, including immunohistochemistry (IHC), fluorescence in situ hybridization (FISH), polymerase chain reaction (PCR)-based assays for point mutations, and, more recently, next-generation sequencing (NGS), which has become increasingly important due to its ability to simultaneously detect multiple genomic alterations in a single assay [10]. As a result, the development and regulatory approval of multiple tyrosine kinase inhibitors (TKIs) have significantly improved survival and disease control in selected patient populations. In addition, assessment of programmed death-ligand 1 (PD-L1) expression by immunohistochemistry has become essential for guiding immunotherapy decisions and serves as an important complement to genomic profiling [10].

**Figure 1:**
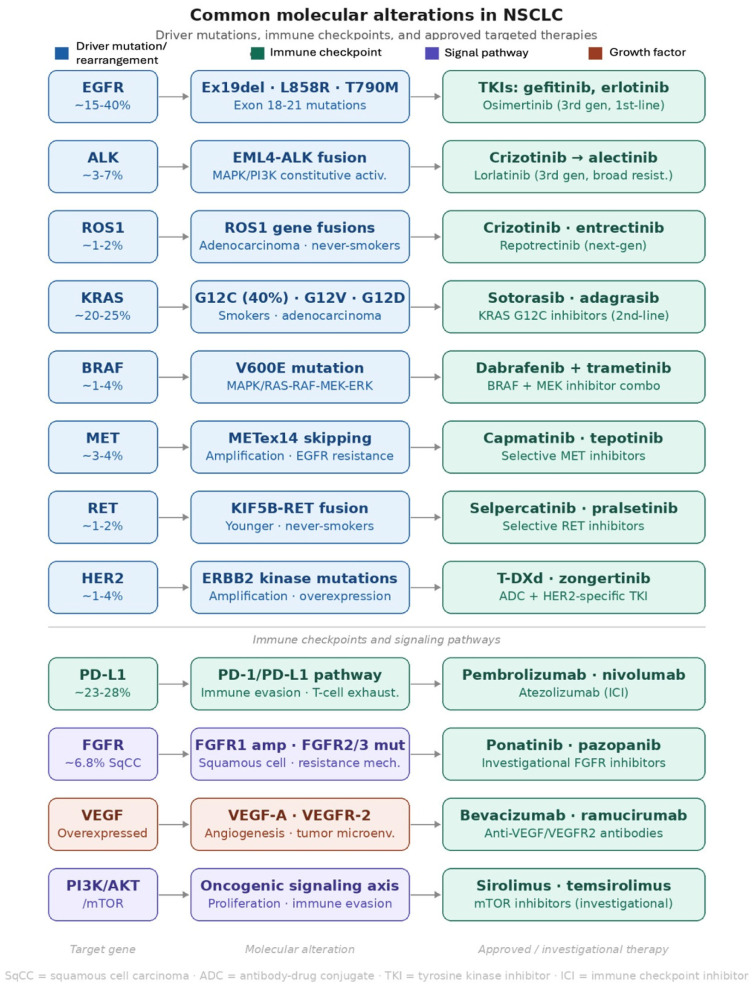
Common molecular alterations in non-small cell lung cancer (NSCLC). Overview of key oncogenic driver mutations, immune checkpoint pathways, and signaling pathway alterations with their approximate prevalence and approved or investigational targeted therapies. Blue boxes indicate driver mutations and rearrangements (EGFR, ALK, ROS1, KRAS, BRAF, MET, RET, HER2); green boxes indicate immune checkpoint pathways (PD-L1); purple boxes indicate signaling pathways (FGFR, PI3K/AKT/mTOR); and coral boxes indicate growth factor pathways (VEGF). Therapy boxes (teal) show currently approved agents or investigational compounds under active clinical evaluation. ADC, antibody–drug conjugate; ICI, immune checkpoint inhibitor; SqCC, squamous cell carcinoma; TKI, tyrosine kinase inhibitor.

The common molecular alterations in NSCLC (Figure 1) include:

### 2.1. EGFR

The epidermal growth factor receptor (EGFR) is a transmembrane receptor tyrosine kinase that plays a critical role in regulating cellular proliferation, survival, differentiation, and migration. Dysregulation of EGFR signaling can promote oncogenesis through persistent activation of downstream pathways involved in tumor growth and progression. In the late 1980s, EGFR overexpression was identified in several malignancies and was subsequently recognized as an important oncogenic driver in NSCLC. Activating EGFR mutations represent one of the most common oncogenic alterations in NSCLC, particularly in patients with adenocarcinoma histology [11,12].

The most frequent activating EGFR mutations are deletions in exon 19 (Ex19del) and the exon 21 L858R point mutation. These mutations account for the majority of EGFR-mutated NSCLC cases and are well established predictive biomarkers for a favorable response to EGFR-TKIs [12]. In addition to these classical mutations, a variety of rare EGFR alterations have been described, including point mutations, insertions, deletions, and duplications within exons 18–25. These rare variants are less common and often demonstrate heterogeneous sensitivity to EGFR-targeted therapies. Due to their rarity, clinical evidence guiding optimal treatment strategies for these mutations remains limited [12].

Three generations of EGFR-TKIs have been developed, resulting in substantial improvements in clinical outcomes compared with conventional cytotoxic chemotherapy [11,13]. First-generation EGFR-TKIs, including gefitinib and erlotinib, reversibly inhibit the ATP-binding site of the EGFR tyrosine kinase domain. These agents demonstrated significantly improved progression-free survival and response rates compared with chemotherapy, particularly in tumors harboring exon 19 deletions or the exon 21 L858R mutation [13]. Second-generation EGFR-TKIs, such as afatinib and dacomitinib, were developed as irreversible inhibitors designed to overcome resistance mechanisms associated with first-generation TKIs. However, their clinical utility has been limited by increased toxicity and limited efficacy against tumors harboring the T790M resistance mutation [14,15,16]. Third-generation EGFR-TKIs, most notably osimertinib, were specifically developed to target both sensitizing EGFR mutations and the T790M resistance mutation while sparing wild-type EGFR, thereby reducing treatment-related toxicity. Clinical trials have demonstrated significant improvements in progression-free survival and overall survival with osimertinib compared with earlier-generation TKIs or chemotherapy [17,18]. Currently, third-generation EGFR-TKIs represent the standard first-line therapy for patients with advanced EGFR-mutated NSCLC. Emerging combination strategies, including the use of third-generation EGFR-TKIs with chemotherapy or novel agents such as amivantamab, have demonstrated promising improvements in progression-free survival and are under active investigation [19].

### 2.2. ALK Rearrangements

ALK rearrangements were first identified in anaplastic large-cell lymphomas and later recognized as an important oncogenic driver in NSCLC, occurring in approximately 3–7% of cases [14,20,21]. In ALK-rearranged NSCLC, fusion proteins such as EML4–ALK lead to constitutive activation of downstream signaling pathways, including MAPK, PI3K–AKT–mTOR, and JAK–STAT, promoting tumor cell proliferation, survival, and progression [21].

ALK tyrosine kinase inhibitors (ALK-TKIs) have significantly improved clinical outcomes compared with chemotherapy, with higher response rates, prolonged progression-free survival (PFS), and better control of central nervous system (CNS) metastases [22]. However, acquired resistance inevitably develops, mainly through secondary ALK mutations, different fusion variants, or activation of bypass signaling pathways [20,23].

Multiple generations of ALK inhibitors have been developed to overcome resistance. First-generation crizotinib showed superior efficacy compared with chemotherapy but is limited by poor CNS penetration and resistance [24]. Second-generation inhibitors (ceritinib, alectinib, brigatinib, ensartinib) offer improved potency and intracranial activity and are widely used as first-line treatments [25,26]. The third-generation inhibitor lorlatinib was designed to overcome a broad range of resistance mutations, including G1202R, which confers resistance to earlier-generation ALK inhibitors [27].

### 2.3. ROS1 Rearrangements

ROS1 gene fusions represent oncogenic drivers in NSCLC, resulting from chromosomal translocations that generate constitutively active fusion proteins, leading to persistent activation of downstream signaling pathways, including MAPK, PI3K, and JAK/STAT. Activation of these pathways promotes tumor growth, survival, and disease progression. ROS1 rearrangements occur in approximately 1–2% of NSCLC cases and are most commonly observed in patients with adenocarcinoma histology, particularly in younger individuals and never-smokers [14,28]. Studies suggest that ROS1 rearrangements may be more frequently observed in female patients and in those presenting with advanced-stage disease (stage III–IV) [29]. NGS is essential for detecting known and novel ROS1 fusion variants with potential therapeutic implications [30,31].

Crizotinib, a multitargeted TKI targeting MET, ALK, and ROS1, was the first approved targeted therapy for ROS1-rearranged NSCLC [32], achieving objective response rates (ORR) of approximately 72% and a median PFS of 19.2 months in patients with ROS1-positive NSCLC [33]. Newer ROS1 inhibitors have been developed to improve treatment outcomes, particularly with regard to CNS activity. Agents such as ceritinib, entrectinib, and lorlatinib demonstrate improved CNS penetration and have become important components of therapeutic strategies for ROS1-rearranged NSCLC [32]. More recently, next-generation ROS1 inhibitors have been introduced to address resistance mutations. Repotrectinib, a potent ROS1/ALK/TRK inhibitor, retains activity against resistance mutations, including the solvent-front G2032R mutation. Clinical studies have demonstrated promising efficacy, with an ORR of 79% (95% CI, 68–88) and a median PFS of 34.1 months in patients with ROS1 fusion-positive NSCLC [34,35]. Another emerging agent is taletrectinib, a CNS-penetrant ROS1/TRK inhibitor that has demonstrated encouraging clinical activity, with an objective response rate of approximately 54% and a median PFS of 9.6 months in patients with advanced or metastatic ROS1-positive NSCLC [36].

### 2.4. BRAF Mutations

BRAF mutations, particularly the BRAF V600E mutation, are oncogenic alteration in NSCLC that leads to constitutive activation of the RAS–RAF–MEK–ERK signaling cascade, also known as the MAPK pathway, promoting uncontrolled cellular proliferation, survival, and tumor progression [37,38]. These alterations are most frequently observed in lung adenocarcinoma and has been reported to occur more commonly in female patients. In addition, co-occurring genomic alterations have been described in some cases, including mutations involving other oncogenic drivers such as EGFR, ALK, and ROS1. The presence of such concurrent mutations may influence disease behavior and therapeutic decision-making [39].

Targeted therapies directed against BRAF V600E mutations have been developed for the treatment of advanced NSCLC. The combination of dabrafenib, a BRAF inhibitor, and trametinib, a MEK inhibitor, has demonstrated significant antitumor activity in patients with BRAF V600E-mutated NSCLC. Clinical studies have shown that this dual-targeted approach improves treatment outcomes compared with conventional chemotherapy while maintaining a manageable safety profile in patients with metastatic disease [37,40].

### 2.5. Kirsten Rat Sarcoma Viral Oncogene Homolog (KRAS)

Mutations in KRAS represent the most common oncogenic driver in NSCLC, accounting for approximately 20–25% of cases. KRAS mutations are predominantly observed in lung adenocarcinoma and are strongly associated with tobacco exposure, occurring in up to 90% of smokers with NSCLC [41]. Among KRAS-mutated tumors, the most frequent subtype is KRAS G12C, which accounts for approximately 40% of KRAS mutations, followed by G12V (19%) and G12D (15%) [42]. Historically, KRAS mutations were considered “undruggable” due to the absence of suitable binding pockets for small-molecule inhibitors. However, recent advances in molecular drug design have enabled the development of targeted therapies specifically directed against KRAS mutations, particularly KRAS G12C, marking a major breakthrough in the treatment of KRAS-mutated NSCLC [43].

Sotorasib and adagrasib have been developed as selective small-molecule inhibitors targeting KRAS G12C mutations. These agents specifically bind to the mutant KRAS protein and prevent its transition to the active guanosine triphosphate (GTP)-bound state, thereby inhibiting downstream oncogenic signaling pathways [44]. Both drugs have been approved and are widely used as second-line treatments for patients with advanced NSCLC harboring KRAS G12C mutations [14], and have demonstrated meaningful clinical activity with these agents. In patients with NSCLC treated with sotorasib, the ORR was 37% (95% CI: 28.6–46.2), with some responses lasting longer than 12 months. The median PFS was 6.8 months (95% CI: 5.1–8.2), and the median overall survival was 12.5 months (95% CI: 10.0–not estimable) [45,46].

Resistance to KRAS inhibitors remains a significant clinical challenge. Mechanisms of resistance may involve genetic alterations affecting nucleotide exchange function, adaptive activation of downstream signaling pathways, or the emergence of additional KRAS mutations, including newly expressed KRAS G12C variants [42]. Furthermore, the presence of co-occurring genomic alterations can influence therapeutic response to KRAS-targeted therapies. For example, mutations in STK11 and ATM frequently co-occur with KRAS mutations such as G12C, G12A, and G12V, while KEAP1 alterations are commonly associated with KRAS G12C and G13X mutations [44].

### 2.6. Human Epidermal Growth Factor Receptor 2 (HER2) Alterations

Human epidermal growth factor receptor 2 (HER2, also known as ERBB2) is a member of the ERBB family of receptor tyrosine kinases. Genetic alterations involving the ERBB2 gene, located on chromosome 17, occur in approximately 1–4% of NSCLC cases [47]. These alterations may include activating mutations, most commonly within the kinase domain (exons 18–21), as well as gene amplification and protein overexpression [48].

Trastuzumab deruxtecan, an antibody–drug conjugate targeting HER2, has been approved for patients with advanced NSCLC harboring HER2 mutations who have previously received systemic therapy. In addition, newer HER2-specific TKIs, such as zongertinib and BAY2927088, have demonstrated promising clinical activity and are emerging therapeutic options for patients with ERBB2-mutated advanced NSCLC demonstrating ORR of 72% and a disease control rate of 95.5%, along with a favorable safety profile [49,50].

### 2.7. Mesenchymal–Epithelial Transition (MET) Alterations

Alterations in the mesenchymal–epithelial transition (MET) gene, located on chromosome 7q21–q31, can lead to constitutive activation of the MET receptor tyrosine kinase, promoting cellular proliferation, survival, and tumor metastasis. The most clinically relevant MET alterations include MET exon 14 skipping mutations (METex14) and MET gene amplification [51]. MET exon 14 skipping mutations occur in approximately 3–4% of lung adenocarcinomas and 1–2% of other NSCLC subtypes. These alterations are particularly enriched in pulmonary sarcomatoid carcinoma, where they are detected at a higher frequency [52]. Patients with METex14-mutated NSCLC tend to be older compared with those harboring other oncogenic driver mutations such as EGFR or KRAS, with a reported median age of approximately 72.5 years at diagnosis [53]. In addition to acting as a primary oncogenic driver, MET amplification has also been identified as an important mechanism of acquired resistance to targeted therapies, particularly EGFR TKIs. MET amplification has been detected in approximately 21% of patients who develop resistance to first-generation EGFR inhibitors such as gefitinib or erlotinib [54,55].

Several targeted therapies have demonstrated clinical efficacy in NSCLC patients harboring MET exon 14 skipping mutations. Crizotinib, a multitargeted kinase inhibitor, as well as more selective MET inhibitors such as capmatinib and tepotinib, have shown significant therapeutic activity in this patient population [56]. Clinical trials have demonstrated encouraging outcomes with these agents. Capmatinib achieved an ORR of 68% (95% CI, 48–84) and a median duration of response of 12.6 months in treatment-naive patients with METex14-mutated NSCLC [57]. Similarly, tepotinib demonstrated an ORR of 57.3% (95% CI, 49.4–65.0) with a median duration of response of 46.4 months in patients with METex14-mutated NSCLC in long-term follow-up analyses [58].

### 2.8. RET Rearrangements

The RET proto-oncogene, located on chromosome 10q11.2, encodes a receptor tyrosine kinase involved in the regulation of cellular growth, differentiation, and survival. RET fusions were first identified as oncogenic drivers in NSCLC in 2011 and occur in approximately 1–2% of NSCLC cases, predominantly in patients with lung adenocarcinoma, younger age, and minimal or no smoking history [59,60]. The most common RET fusion partner identified in NSCLC is KIF5B–RET, while less frequent fusion partners include CCDC6–RET and NCOA4–RET [61].

Selpercatinib and pralsetinib—selective RET inhibitors—have demonstrated substantial antitumor activity with favorable safety profiles in patients with advanced disease [62,63]. In patients with advanced RET fusion-positive NSCLC, selpercatinib showed significant clinical benefit, achieving an ORR of 64% and a median duration of response of 17.5 months [64]. Similarly, pralsetinib has demonstrated promising efficacy, with an ORR of 72% and a median progression-free survival (PFS) of 13.0 months in treatment-naïve patients [65].

### 2.9. Fibroblast Growth Factor Receptor (FGFR) Alterations

FGFR alterations represent oncogenic drivers in a subset of NSCLC. The FGFR signaling pathway plays an important role in regulating cell proliferation, survival, differentiation, and angiogenesis, thereby contributing to tumor development and progression [66,67]. Activation of the FGFR pathway has also been implicated as a compensatory resistance mechanism during treatment with targeted therapies, including EGFR- or KRAS-directed therapies [68]. FGFR alterations are most commonly observed in lung squamous cell carcinoma, where FGFR mutations occur in approximately 6.8% of cases, whereas they are less frequent in other NSCLC subtypes, with a reported prevalence of approximately 1.3% [66]. Several types of FGFR alterations have been identified in NSCLC, including FGFR1 amplification, FGFR2 and FGFR3 mutations, and FGFR gene fusions. Examples of reported fusion events include FGFR3–TACC3, FGFR2–INA, FGFR4–RAPGEFL1, and FGFR1 fusions [66,69].

Targeted therapies directed against FGFR, e.g., ponatinib, have demonstrated inhibitory effects on tumor growth in NSCLC cell lines overexpressing FGFR1 [70]. In addition, multikinase inhibitors, such as pazopanib, have shown clinical responses in patients with FGFR2-mutated squamous cell carcinoma [71]. However, the clinical effectiveness of FGFR-targeted therapies may be limited by the development of acquired resistance, which can arise from secondary mutations within the FGFR kinase domain. Moreover, FGFR fusions may emerge as mechanisms of resistance to other targeted treatments, including EGFR tyrosine kinase inhibitors. Therefore, comprehensive genomic profiling, including circulating tumor DNA (ctDNA) analysis, is essential for detecting FGFR alterations and guiding personalized treatment strategies in NSCLC [69,72].

### 2.10. Trophoblast Cell Surface Antigen 2 (TROP-2) Alterations

Trophoblast cell surface antigen 2 (TROP-2) is a transmembrane glycoprotein belonging to the epithelial cell adhesion molecule (EpCAM) family and is highly expressed in several epithelial malignancies, including NSCLC [73]. TROP-2 expression has been reported in approximately 75% of squamous cell carcinomas, 65% of lung adenocarcinomas, and 18% of high-grade neuroendocrine tumors. Furthermore, increased TROP-2 expression in lung adenocarcinoma has been associated with higher cancer-specific mortality, suggesting a potential prognostic role for this biomarker in lung cancer [74].

Targeted therapies against TROP-2 have recently emerged. Sacituzumab govitecan (SG), an antibody–drug conjugate linking an anti-TROP-2 monoclonal antibody to SN-38 (the active metabolite of irinotecan), has demonstrated promising activity in advanced NSCLC, with an ORR of 17% in the intention-to-treat population (19% in evaluable patients), a median duration of response of 6.0 months, and a median PFS of 5.2 months [75].

### 2.11. Other Molecular Pathways in NSCLC

#### 2.11.1. Programmed Cell Death Protein-1 (PD-1) Receptor and Programmed Cell Death Ligand-1 (PD-L1) Pathway

The programmed cell death protein 1 (PD-1) receptor and its ligands PD-L1 and PD-L2 constitute an important immune checkpoint pathway that plays a key role in tumor immune evasion. PD-1 is a co-inhibitory receptor primarily expressed on activated T-cells, B cells, natural killer (NK) cells, monocytes, and dendritic cells (DCs). Activation of the PD-1/PD-L1 pathway contributes to immune suppression within the tumor microenvironment and facilitates tumor immune escape in NSCLC [76,77]. The interaction between PD-1 and PD-L1/PD-L2 inhibits T-cell activation and proliferation, reduces the cytotoxic function of T lymphocytes, and promotes T-cell exhaustion. These effects impair the host immune response against tumor cells and allow malignant cells to evade immune surveillance. Additionally, regulatory mediators such as GATA-3 and T-bet have been implicated in promoting apoptosis of tumor-infiltrating T-cells, further contributing to an immunosuppressive tumor microenvironment [77].

Therapeutic blockade of the PD-1/PD-L1 pathway is a cornerstone in advanced NSCLC management, restoring antitumor immunity and enhancing T-cell-mediated tumor cell killing. Approved inhibitors, including pembrolizumab, nivolumab, and atezolizumab, have demonstrated improved survival, durable responses, and manageable safety profiles in first- and second-line settings [76,78].

#### 2.11.2. Cytotoxic t-Lymphocyte-Associated Antigen 4 (CTLA-4) Pathway

Cytotoxic T-lymphocyte-associated antigen 4 (CTLA-4) is another important immune checkpoint protein involved in the regulation of immune responses. Similar to PD-1, CTLA-4 functions as a negative regulator of T-cell activation, playing a critical role in maintaining immune homeostasis and self-tolerance. By inhibiting early T-cell activation, CTLA-4 contributes to the suppression of antitumor immune responses [79]. In NSCLC, the role of CTLA-4 expression remains complex and has been associated with both prognostic and predictive significance. Studies have also shown that CTLA-4 expression may differ between primary tumors and metastatic lymph nodes, reflecting phenotypic heterogeneity within the tumor microenvironment [80].

Therapeutic inhibition of CTLA-4 enhances T-cell activation and restores antitumor immunity. While agents such as tremelimumab have been evaluated in advanced NSCLC, CTLA-4 blockade is primarily used in combination with PD-1/PD-L1 inhibitors, where dual checkpoint inhibition has shown improved immune responses, more durable clinical benefit, and acceptable safety profiles [81,82].

#### 2.11.3. The Phosphatidylinositol 3-Kinase (PI3K)/AKT/Mammalian Target of Rapamycin (mTOR) Signaling Pathway

The phosphatidylinositol 3-kinase (PI3K)/AKT/mammalian target of rapamycin (mTOR) signaling pathway plays a critical role in tumorigenesis and disease progression and represents a central oncogenic axis in NSCLC. Activation of this pathway promotes tumor growth through multiple mechanisms, including enhanced cellular proliferation, metabolic reprogramming, inhibition of apoptosis, and increased resistance to anticancer therapies [83,84]. Activation of the PI3K/AKT/mTOR pathway has also been implicated in shaping the tumor immune microenvironment. Dysregulation of this pathway in tumor or immune cells may contribute to the development of an immunosuppressive tumor microenvironment, which facilitates tumor progression and immune evasion. In addition, activation of this signaling cascade has been associated with increased expression of immune checkpoint molecules such as PD-L1, further promoting immune escape in NSCLC [84,85]. Furthermore, activation of the PI3K/AKT/mTOR signaling pathway plays an important role in metabolic regulation, particularly by enhancing glycolytic activity in tumor cells. This metabolic reprogramming contributes to increased tumor growth and progression in NSCLC [86]. Numerous inhibitors targeting PI3K, AKT, and mTOR are currently under development and are being evaluated in preclinical studies and early-phase clinical trials for the treatment of NSCLC [84]. Examples of agents targeting this pathway include perifosine, an alkylphospholipid compound that inhibits AKT membrane translocation and prevents phosphorylation of downstream signaling molecules, thereby disrupting oncogenic signaling pathways. Other agents under clinical investigation include sirolimus, temsirolimus, and ridaforolimus, which target components of the mTOR signaling pathway [84,87].

#### 2.11.4. Vascular Endothelial Growth Factor (VEGF) Pathway

The vascular endothelial growth factor (VEGF) signaling pathway plays a critical role in the progression, metastasis, and recurrence of NSCLC. VEGF mediates the formation of tumor microvasculature through angiogenesis, the process by which new blood vessels are formed to supply oxygen and nutrients to rapidly proliferating tumor cells. This process facilitates tumor growth, invasion, and metastatic dissemination. Many NSCLC tumors exhibit overexpression of VEGF-A, which represents a key driver of tumor-associated angiogenesis [88,89,90]. In addition to its role in angiogenesis, VEGF significantly influences the tumor immune microenvironment. VEGF can inhibit the maturation of dendritic cells, impair antigen-presenting capacity, and promote the recruitment of regulatory T-cells, tumor-associated macrophages, and myeloid-derived suppressor cells, thereby contributing to an immunosuppressive tumor microenvironment that facilitates tumor immune evasion [88]. The VEGF family includes several ligands such as VEGF-A, VEGF-B, VEGF-C, VEGF-D, and placenta growth factor (PlGF). These ligands interact with specific receptors, including VEGFR-1, VEGFR-2, and VEGFR-3, as well as co-receptors such as neuropilin-1 (NRP-1) and neuropilin-2 (NRP-2), to regulate both angiogenesis and lymphangiogenesis [91].

Targeting the VEGF/VEGFR signaling axis has become an important therapeutic strategy in NSCLC. Several agents have been developed, including monoclonal antibodies directed against VEGF or VEGF receptors as well as receptor tyrosine kinase inhibitors. Among these, bevacizumab, a monoclonal antibody targeting VEGF-A, and ramucirumab, a human IgG1 monoclonal antibody targeting VEGFR-2, have demonstrated clinical efficacy when combined with chemotherapy in patients with advanced NSCLC, leading to improved response rates, progression-free survival, and overall survival [88,90,92]. More recently, combination strategies integrating anti-angiogenic therapy with immune checkpoint inhibitors have shown promising results. Anti-angiogenic agents can normalize tumor vasculature and modulate the tumor microenvironment, shifting it from an immunosuppressive to an immune-supportive state, thereby enhancing the efficacy of immunotherapy [93]. Additionally, serum VEGF levels have been investigated as a potential predictive biomarker in NSCLC. However, further research is required to clarify its clinical utility for diagnosis, prognosis, and treatment selection [91].

## 3. Importance of Mediastinal Staging and Tissue Acquisition

Accurate staging of NSCLC is essential for determining optimal management strategies and for estimating prognosis, including survival outcomes and the risk of disease recurrence. Furthermore, precise staging also plays a critical role in guiding therapeutic decision-making, including the selection of the surgical resection, systemic therapies, radiotherapy, or combined multimodal treatment approaches [94]. The tumor–node–metastasis (TNM) staging system, established by the International Association for the Study of Lung Cancer (IASLC), is the globally accepted standard classification for lung cancer staging [94].

The IASLC released the 9th edition of the TNM classification for lung cancer in January 2025, introducing several revisions aimed at improving prognostic accuracy and supporting increasingly complex treatment strategies NSCLC [95]. In the 8th edition, N categories were defined exclusively according to the anatomical location of metastatic lymph nodes, without considering the number of involved lymph nodes. However, growing evidence has demonstrated that additional factors beyond anatomical location contribute to significant prognostic heterogeneity, particularly within the broad N2 category, highlighting the need for a more refined classification system [96]. While the T category underwent minor anatomical refinements, it remained largely unchanged in its fundamental structure. The most significant modifications involve the nodal and metastatic classifications. Specifically, pathological N2 disease has been subdivided into two categories: N2a and N2b, reflecting differences in nodal disease burden. In addition, the M1c category has been further subdivided into M1c1 and M1c2, providing a more detailed classification of distant metastatic spread [94,95]. These updates emphasize the importance of pathological nodal staging, which remains one of the most important prognostic factors in patients with resectable NSCLC. Furthermore, emerging evidence suggests that N2b disease is associated with a significantly higher risk of mortality compared with N2a disease, supporting the clinical relevance of this more granular nodal classification. Overall, the refinements introduced in the 9th edition of the TNM staging system are expected to improve the accuracy of prognostic assessment, facilitate more precise patient stratification, and enhance clinical decision-making in the management of NSCLC [96].

### 3.1. Clinical Importance of Comprehensive Mediastinal Lymph Node Staging in Early-Stage NSCLC

Systematic and accurate lymph node staging in early-stage NSCLC planned for curative surgical management is essential to ensure precise disease staging, guide appropriate adjuvant therapy, and achieve complete (R0) resection at the time of surgery [97]. Inadequate resection—whether resulting from incomplete lymph node dissection (e.g., failure to perform systematic nodal dissection or the presence of a positive highest mediastinal lymph node) or from an uncertain resection status—has been associated with significantly worse overall survival compared with complete surgical resection (hazard ratios 1.69 and 3.18, respectively; both *p* = 0.0001). Median overall survival was 80.1 months in patients with complete resection, compared with 39.9 months and 17.3 months in those with uncertain and incomplete resections, respectively. Corresponding 5-year survival rates were 58.8%, 37.3%, and 15.7%, respectively. These findings underscore the critical importance of accurate mediastinal nodal staging [98].

### 3.2. Clinical Importance of Comprehensive Mediastinal Lymph Node Staging in Locally Advanced NSCLC

In locally advanced NSCLC, accurate mediastinal nodal staging is essential for appropriate radiotherapy planning. Reliance solely on PET-CT for determining the extent of nodal involvement may lead to inaccuracies in target volume delineation. False-positive lymph nodes on PET-CT may result in unnecessarily enlarged radiation fields and higher delivered radiation doses, thereby increasing the risk of treatment-related toxicity and complications. Conversely, PET-occult nodal metastases may be excluded from the radiation field, which can increase the risk of geographic miss, treatment failure, and disease recurrence [99]. Current evidence suggests that a considerable proportion of patients may harbor nodal disease not detected by PET-CT alone. In particular, patients with larger primary tumors and PET-CT-based clinical N0 or N1 status demonstrate a higher likelihood of occult mediastinal involvement [99]. Studies have reported upstaging to pathological N2 disease in approximately 20% of such patients, identified either through endobronchial ultrasound-guided transbronchial needle aspiration (EBUS-TBNA) or postoperative pathological assessment [100].

These observations are consistent with estimates indicating that 5–29% of patients may have nodal disease located outside the initially planned radiation field when staging is based solely on imaging modalities. This underscores the importance of incorporating invasive mediastinal staging techniques, such as EBUS-guided nodal sampling, to improve staging accuracy and optimize radiotherapy target delineation in patients with locally advanced NSCLC [97,100,101].

## 4. Role of EBUS-TBNA Samples for Immunohistochemistry and NGS

EBUS-TBNA plays a crucial role in precision oncology by providing cytological and histological material for molecular characterization during lung cancer staging, thereby enabling the identification of patients who may benefit from targeted therapies and personalized immunotherapy [102]. Additionally, the molecular analysis of EBUS-TBNA-derived lymph node specimens may improve nodal staging accuracy through two complementary mechanisms. First, EBUS-TBNA enables simultaneous pathological confirmation of nodal metastasis and comprehensive molecular profiling within a single minimally invasive procedure, thereby integrating anatomical staging with molecular characterization. Second, the detection of tumor-specific oncogenic driver mutations in mediastinal lymph node samples may enhance the sensitivity of nodal staging beyond conventional cytomorphological assessment, particularly in cases of occult or micrometastatic nodal involvement where standard cytological evaluation may be insufficient [103,104].

The adequacy and reliability of EBUS-TBNA for genotyping in NSCLC, as well as its suitability for immunohistochemical analysis, have been widely investigated [105]. In a large multicenter study including 774 patients, the suitability of EBUS-TBNA specimens obtained in routine clinical practice for NSCLC subtyping and molecular analysis was evaluated. Histological subtyping was successfully achieved in 77% of cases, while EGFR mutation analysis was feasible in 90% of the obtained samples. These findings support the role of EBUS-TBNA as a reliable minimally invasive technique for both histological classification and molecular testing in patients with NSCLC [106].

Similarly, a retrospective analysis of 109 patients with NSCLC and mediastinal or hilar lymphadenopathy demonstrated the feasibility of using EBUS-TBNA specimens for the detection of EML4–ALK fusion genes. ALK rearrangements were identified in 6.4% of cases using EBUS-TBNA samples. These alterations were reliably detected by IHC, FISH, and reverse transcription polymerase chain reaction (RT-PCR), demonstrating that small histological core samples obtained via EBUS-TBNA are suitable for comprehensive molecular analysis. ALK-positive tumors were associated with adenocarcinoma histology, younger age, never- or light-smoking status, smaller primary tumor size, mucin production, and wild-type EGFR [107].

The feasibility and reliability of EBUS-TBNA samples for PD-L1 assessment and broad molecular characterization in advanced NSCLC were further evaluated in a prospective study including 42 patients. EBUS-TBNA samples showed high adequacy for comprehensive molecular profiling, with successful genotyping rates of 92.8% using NGS, 95.2% using nCounter analysis, and 100% for PD-L1 testing. Concordance with paired tissue biopsy samples was 100% for NGS and nCounter analyses and substantial for PD-L1 expression (88.9% using a ≥ 1% cutoff), although caution is warranted when interpreting very high PD-L1 expression levels [102].

A systematic review and meta-analysis including 33 studies and 2869 patients further confirmed the high adequacy of EBUS-TBNA samples for molecular testing in NSCLC. The pooled sample adequacy was 94.5% (95% CI, 93.2–96.4%) for EGFR mutation analysis and 94.9% (95% CI, 89.4–98.8%) for ALK rearrangement testing. The prevalence of EGFR mutations was 15.8% (95% CI, 12.1–19.4%), whereas ALK rearrangements were detected in 2.77% of cases (95% CI, 1.0–4.8%). Data regarding ROS1 and PD-L1 alterations were insufficient for meta-analysis [108].

### 4.1. Factors Influencing the Adequacy and Diagnostic Yield of EBUS-TBNA Samples for Molecular Mutation Analysis

**Figure 2:**
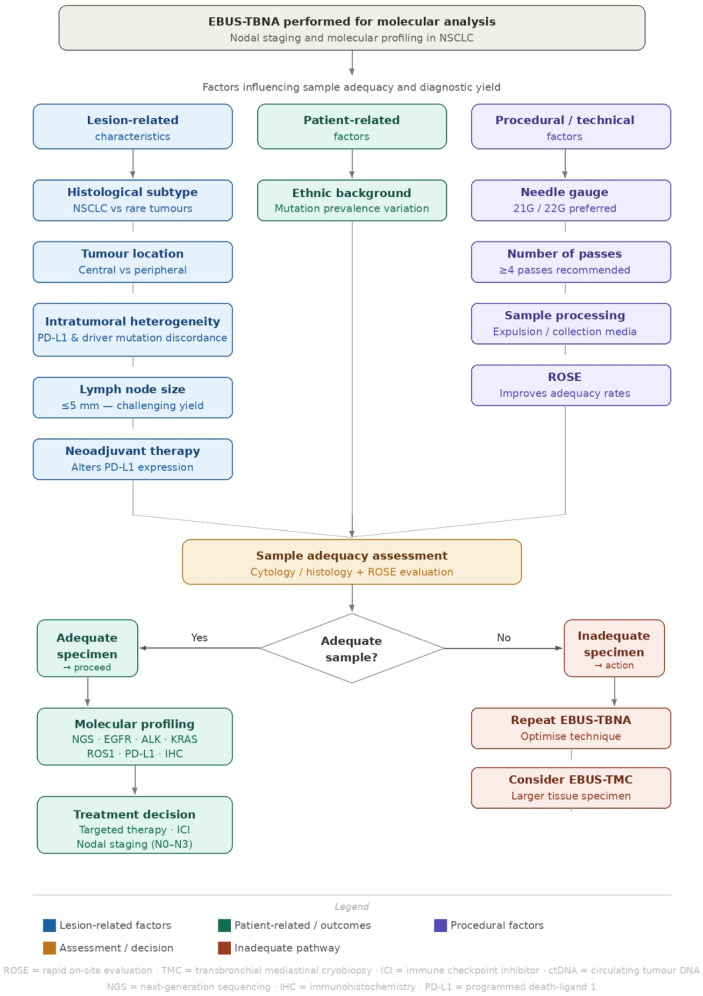
Algorithm illustrating factors influencing the adequacy and diagnostic yield of EBUS-TBNA samples for molecular mutation analysis in NSCLC. EBUS-TMC: EBUS-guided transbronchial mediastinal cryobiopsy; ICI: immune checkpoint inhibitor; IHC: immunohistochemistry; NGS: next-generation sequencing; NSCLC: non-small cell lung cancer; PD-L1: programmed death-ligand 1; ROSE: rapid on-site evaluation.

Multiple factors influence the diagnostic accuracy and reliability of molecular analyses performed on samples obtained via EBUS-TBNA (Figure 2). These determinants can be broadly categorized into three principal domains: (a) lesion-related characteristics: these include the histological subtype of the tumor, anatomical tumor location, the presence of intratumoral heterogeneity, the size of metastatic lymph nodes, and prior neoadjuvant therapy, all of which may affect cellular yield and the representativeness of the obtained specimen [105,109,110], (b) patient-related factors: patient characteristics, particularly the ethnic background of the studied population, may influence the prevalence and distribution of specific molecular alterations [108], (c) procedural and technical factors of EBUS-TBNA: technical aspects of the sampling procedure significantly affect specimen adequacy and molecular testing success. Relevant parameters include the needle gauge, the application of suction, the number of needle passes, specimen handling and processing protocols, and the utilization of rapid on-site evaluation (ROSE) to assess sample adequacy during the procedure [111].

#### 4.1.1. Lesion-Related Characteristics

##### Histological Subtype of Lung Cancer

The histological subtype of lung cancer represents an important factor influencing the accuracy and reliability of molecular analyses performed on samples obtained by EBUS-TBNA [111]. In patients with primary NSCLC, molecular testing for common driver mutations—such as EGFR, KRAS, BRAF, and PIK3CA—can be reliably performed on EBUS-TBNA specimens. Studies have demonstrated a high feasibility of mutation analysis, with an overall mutation detection rate of approximately 91% using fine-needle aspirates analyzed by allele-specific qPCR [112]. In contrast, the diagnostic yield of EBUS-TBNA samples may be limited in rare tumors or uncommon malignancies, where small cytological specimens are often insufficient for comprehensive histopathological evaluation and advanced molecular profiling. In such cases, larger tissue samples are frequently required to establish a definitive diagnosis and to perform extended molecular analyses. Alternative techniques, such as EBUS-guided transbronchial mediastinal cryobiopsy (TMC), may provide larger and more representative tissue specimens, thereby improving diagnostic accuracy in patients with mediastinal lymphadenopathy without an obvious primary lung lesion [112,113].

##### Location of the Tumor

The location of the tumor may also influence the diagnostic accuracy and reliability of samples obtained by EBUS-TBNA. Specimens acquired from centrally located intrapulmonary tumors have demonstrated diagnostic performance comparable to that of mediastinal lymph node sampling and frequently provide sufficient material for molecular analysis [114]. EBUS-TBNA performed on centrally located tumors achieved an overall diagnostic yield of 86% (88/102 cases). Furthermore, the adequacy of samples for molecular testing was reported in ≥94% of cases [114].

##### Intratumoral Heterogeneity

PD-L1 expression in NSCLC demonstrates both intertumoral and intratumoral heterogeneity, which may contribute to discordance in PD-L1 classification between small cytological specimens, such as those obtained by EBUS-TBNA, and larger surgically resected tissue samples. This variability may affect the reliability of PD-L1 assessment, particularly when tumor proportion score (TPS) values are close to clinically relevant cut-off thresholds of 1% and 50% [115]. Furthermore, discordant PD-L1 expression between different tumor sites or specimen types is not uncommon in NSCLC, reflecting heterogeneity related to histological subtype, tissue sampling methods, and metastatic sites [116].

##### Size of Metastatic Lymph Nodes

The size of metastatic lymph nodes may also influence the reliability of molecular analyses performed on EBUS-TBNA-derived specimens, although the impact of lymph node size remains controversial [117]. Larger lymph nodes are generally more likely to harbor metastatic disease and therefore may provide a higher probability of obtaining malignant cells in sufficient quantity for histopathological assessment and molecular profiling [117,118]. Conversely, smaller lymph nodes, particularly those measuring ≤ 5 mm, are less frequently associated with malignancy and may pose technical challenges during sampling, potentially resulting in insufficient material for molecular analysis [119].

However, some studies suggest that lymph node size may not significantly influence diagnostic performance. For example, a prospective study showed that diagnostic accuracy of EBUS-TBNA was not significantly associated with lymph node size or the number of needle passes (*p* = 0.23 and *p* = 0.27, respectively), indicating that adequate diagnostic yield can still be achieved even in smaller lymph nodes when appropriate technique is applied [117].

##### Neoadjuvant Chemotherapy or Immunotherapy

Neoadjuvant therapy, including chemotherapy or immunotherapy, may also influence the expression of PD-L1 in tumor cells. Several studies have suggested that systemic treatment can alter the tumor microenvironment and may lead to changes in PD-L1 expression levels, including potential downregulation of PD-L1 on tumor cells. Consequently, PD-L1 assessment performed on post-treatment samples may not always accurately reflect the baseline PD-L1 status, which could affect the interpretation of results obtained from EBUS-TBNA specimens used for molecular and immunohistochemical analysis [120].

#### 4.1.2. Technical Factors of EBUS-TBNA

Sample adequacy in EBUS-TBNA is influenced by several technical factors, including needle size, the use of suction, the number of needle passes, specimen processing protocols, and the integration of rapid on-site evaluation (ROSE) [111].

##### Gauge of EBUS-TBNA Needles

Multiple EBUS-TBNA needle gauges (25-, 22-, 21-, and 19-gauge) are currently commercially available. The optimal needle size for obtaining adequate tissue samples for diagnosis and molecular testing has been investigated in numerous studies. The American College of Chest Physicians (ACCP) Clinical Practice Guideline for the acquisition and handling of endobronchial ultrasound-guided transbronchial needle aspiration samples recommends the use of 21- or 22-gauge needles over 19-gauge needles when performing EBUS-TBNA in patients with suspected malignant disease, mainly due to concerns regarding tissue integrity and potential specimen contamination with blood when using larger needles [111]. However, this recommendation is conditional and based on very low certainty of evidence [111].

Several studies have reported no significant difference in sample adequacy or diagnostic yield between 21-G and 22-G needles. In a comparative study demonstrated that although the diagnostic yield was similar between the two needle sizes, samples obtained with the 21-G needle showed better preservation of histological architecture, which may facilitate improved morphological and genetic characterization of NSCLC [103].

The role of the 19-G needle, which may allow retrieval of a larger tissue core, remains controversial. While the larger needle may theoretically improve tissue acquisition for histological and molecular analysis, available evidence is heterogeneous. One limitation of the 19-G needle is increased blood contamination during aspiration, which may complicate ROSE and potentially reduce diagnostic yield in some cases [111]. Several studies have suggested potential advantages of using a larger-gauge needle. In one study, the 19-G needle demonstrated significantly higher smear cellularity compared with the 22-G needle (32.6% vs. 13.0%, *p* = 0.05) and was associated with improved adequacy rates during ROSE (84.8% vs. 63.0%, *p* = 0.004) [121]. Similarly, another study reported that specimens obtained with a 19-G needle contained significantly greater amounts of tissue and higher numbers of tumor cells per slide, while maintaining a safety profile comparable to that of the 22-G needle [121,122]. In contrast, several randomized and prospective studies have demonstrated no superiority of the 19-G needle in terms of diagnostic yield or histological core procurement [123,124,125]. Conversely, one study reported that the 19-G needle did not improve diagnostic yield and produced more bloody and less adequate samples compared with the 22-G needle. In their study, sample adequacy was significantly higher with the 22-G needle (73% vs. 46%, *p* < 0.001), while the 19-G needle produced significantly more bloody samples (59% vs. 19%, *p* < 0.001). Despite these differences, the overall diagnostic yield remained similar between both needles (95% vs. 93%, *p* = 0.62) [104].

Regarding molecular testing, the available evidence suggests that samples obtained with 21-G and 22-G needles are generally sufficient for molecular analysis, including EGFR, ALK, and PD-L1 testing. EGFR mutation testing was successfully performed in 79 of 80 cases (98.75%), with EGFR mutations detected in 5 of 80 samples (6.3%), while ALK analysis was successfully performed in all tested samples (21/21) [126]. Similarly, a pilot study demonstrated that the 19-G needle achieved high success rates for molecular testing, with success rates of 90% for PD-L1, 90% for EGFR, and 86% for ALK testing in NSCLC samples due to the higher tumor cell content. However, the success rate for ROS-1 testing was lower (67%) [127].

Overall, current evidence indicates that 21-G and 22-G needles provide reliable diagnostic yield and adequate material for molecular testing, whereas the potential advantages of 19-G needles remain inconsistent across studies and require further investigation [111].

##### Number of Needle Passes

According to ACCP clinical practice guideline on the acquisition and handling of EBUS-TBNA Samples, at least four needle passes per lymph node are suggested when malignancy is suspected rather than three or fewer passes, although the quality of evidence supporting this recommendation is considered very low. This recommendation aims to ensure adequate tissue acquisition not only for definitive histopathological diagnosis but also for ancillary testing and molecular analysis. Increasing the number of passes per lymph node may improve the likelihood of obtaining sufficient material for molecular testing without an apparent increase in procedure-related complications [111].

Supporting evidence has been reported in several studies. In a cohort of 453 patients with lung cancer, a mean of 3.4 EBUS-TBNA passes per lesion was sufficient to allow EGFR mutation and ALK fusion gene analysis in 80.4% (201/250) of specimens following routine IHC processing. The authors further demonstrated that performing at least three passes per lesion, particularly in larger lymph nodes, significantly increased the probability of successful lung cancer subtyping and molecular testing using EBUS-TBNA samples [128]. Similarly, one study reported that a median of four passes per lesion was required to achieve adequate molecular profiling in 95.3% of cases when EBUS-TBNA was performed in conjunction with ROSE [129].

##### Specimen Sample Expulsion/Suction and Collection Media

The ACCP clinical practice guideline on the acquisition and handling of EBUS-TBNA samples suggests that either the standard method (e.g., using a stylet or air flush) or alternative techniques such as needle rinsing may be used to expel EBUS-TBNA specimens onto cytopathology slides or directly into liquid collection media (e.g., formalin or alcohol-based solutions). However, the guideline does not recommend one expulsion technique over another, reflecting the limited available evidence [111].

The only aspect that has been formally evaluated in a randomized controlled trial is the use of a stylet during specimen expulsion. In this study, 121 patients with 194 sampled lymph nodes underwent EBUS-TBNA using both with-stylet and without-stylet techniques. The results demonstrated no significant difference in diagnostic yield or specimen quality between the two methods, suggesting that the use of a stylet does not improve diagnostic outcomes [130]. To date, no studies have specifically compared different expulsion techniques (e.g., air flush, stylet expulsion, or needle rinsing) with regard to sample adequacy, diagnostic yield, or reliability for molecular analysis. Most available studies instead focus on comparisons between specimen preparation or collection media (e.g., formalin versus alcohol-based media, direct smears versus liquid-based cytology) [111].

Accordingly, the ACCP guideline does not recommend a specific collection medium (such as formalin, RPMI, saline, phosphate-buffered saline, or alcohol-based preparations). These recommendations remain conditional with very low certainty of evidence, largely due to limited comparative data. Several studies have reported no significant differences in diagnostic yield among different preparation methods or collection media. Furthermore, combining direct cytology smears with cell block preparation has been shown to provide high diagnostic accuracy in EBUS-TBNA samples [111,131,132,133]. Given these limitations in the evidence, the guideline emphasizes the importance of local collaboration between bronchoscopists and cytopathology laboratories to determine the most appropriate specimen expulsion technique, collection medium, and processing method based on local expertise and laboratory requirements [111].

Regarding the suitability of cytological specimens for molecular analysis, studies have demonstrated high reliability. For example, in a study evaluating EGFR mutation testing in EBUS-TBNA cytology samples, paired primary tumor tissue and lymph node cytology specimens from 14 patients with EGFR-mutant lung adenocarcinoma were analyzed. The results showed high concordance between cytological and histological samples, with all but one cytology specimen demonstrating the same mutation profile. These findings support the reliability of cytology smears for real-time PCR-based EGFR mutation testing in NSCLC [134]. Additionally, alcohol-based fixatives have been investigated as an alternative to formalin and paraffin-based fixation methods, which may impair DNA and RNA quality. Several studies have demonstrated that alcohol-based non-crosslinking fixatives provide higher DNA and RNA yields with improved preservation of nucleic acid integrity. Furthermore, these fixatives may enhance the molecular diagnostics by reducing nucleic acid damage and increasing the amount of recoverable genetic material from biopsy specimens [135].

##### ROSE

The ACCP clinical practice guideline on the acquisition and handling of EBUS-TBNA samples recommends the use of ROSE during EBUS-TBNA when available, as it provides immediate cytopathological feedback and preliminary diagnostic information compared with procedures performed without ROSE [111]. Evidence supporting this recommendation has been demonstrated in several studies. A pooled analysis evaluating the diagnostic yield of EBUS-TBNA with versus without ROSE reported diagnostic yields of 78.0% and 71.4%, respectively, corresponding to an odds ratio of 2.35 (95% CI, 1.47–3.74) in favor of ROSE [136,137,138,139,140].

Regarding the influence of ROSE on molecular genotyping, a prospective randomized trial comparing EBUS-TBNA with and without ROSE demonstrated that complete molecular profiling was achieved in 85.7% of patients (108/126). Although the success rate was higher in the ROSE group than in the EBUS-only group (90.8% vs. 80.3%), the difference did not reach statistical significance (*p* = 0.09). Notably, the ROSE group had no samples limited to pathologic diagnosis due to minimal tumor burden (0 vs. 6, *p* = 0.05) and more frequently required only a single biopsy site (58.9% vs. 44.1%, *p* = 0.01) [141].

### 4.2. Concordance of EBUS-TBNA with Surgical Specimens for Molecular Profiling

Although EBUS-TBNA is a minimally invasive technique, accumulating evidence demonstrates that specimens obtained with this method are reliable for molecular analyses, including testing for EGFR, ALK, and KRAS mutations. Several studies have shown a high concordance between molecular profiling results obtained from EBUS-TBNA samples and those derived from surgical resection specimens. A prospective study compared the diagnostic accuracy of EBUS-TBNA and mediastinoscopy for mediastinal staging of lung cancer. The two techniques demonstrated excellent agreement (91%; κ = 0.8; 95% CI, 0.7–0.9). Both methods achieved 100% specificity and positive predictive value. The sensitivity, negative predictive value, and overall diagnostic accuracy were 81%, 91%, and 93% for EBUS-TBNA, and 79%, 90%, and 93% for mediastinoscopy, respectively, with no significant difference in pathological N-stage determination (*p* = 0.78) [142].

Regarding PD-L1 testing, a study evaluated the concordance between PD-L1 expression in small biopsy samples and corresponding surgical specimens. Using a PD-L1 tumor proportion score (TPS) cut-off ≥50%, the study demonstrated a 78% concordance rate with moderate agreement (Cohen’s κ = 0.45) between EBUS-TBNA samples and surgical resection specimens. Although PD-L1 expression in biopsy samples correlated with surgical specimens, the authors noted that the clinical accuracy remains limited, indicating that PD-L1 assessment from small biopsy samples should be interpreted cautiously when guiding treatment decisions [143]. Similar findings were observed 78% concordance with moderate agreement (κ = 0.45) when using a TPS cut-off ≥50% for PD-L1 expression [120].

For driver mutation testing, a study demonstrated that comprehensive molecular profiling can be effectively performed using EBUS-TBNA samples. In their study, EGFR mutations (exons 18–21), KRAS mutations (exons 2–3), and ALK rearrangements were identified in 16.9%, 31.6%, and 3.9% of EBUS-TBNA samples, respectively, compared with 14.8%, 29.0%, and 3.4% in corresponding surgical specimens. Importantly, no statistically significant differences were observed between the two sample types, confirming the suitability of EBUS-TBNA specimens for comprehensive mutational analysis in advanced non-small cell lung cancer [144].

### 4.3. EBUS-Transbronchial Mediastinal Cryobiopsy (TMC) and Molecular Testing

EBUS-TMC has recently emerged as a promising technique for sampling mediastinal and hilar lymph nodes. Compared with conventional needle aspiration, cryobiopsy generally yields larger specimens with well-preserved tissue architecture and minimal crush artifacts, which facilitates histopathological evaluation and may improve the suitability of samples for molecular analyses [145]. Initial clinical experience suggests that the procedure has a favorable safety profile, with no significant difference in the rate of adverse events compared with standard EBUS-TBNA [145,146]. In clinical practice, EBUS-TMC is frequently used as a complementary technique to EBUS-TBNA, improving diagnostic performance when both methods are combined. In a multicenter, open-label randomized trial, the diagnostic yield increased from 81% with EBUS-TBNA alone to 93% when combined with cryobiopsy [145]. EBUS-TMC appears to be particularly valuable in lymphoproliferative disorders and benign granulomatous diseases, such as sarcoidosis. In one study, the diagnostic rate for sarcoidosis reached 96.7% when EBUS-TBNA was combined with cryobiopsy, compared with 87.5% when EBUS-TBNA was combined with transbronchial forceps biopsy [147]. A pooled analysis of seven studies including 555 patients reported an overall diagnostic yield of 92% for EBUS-TMC compared with 80% for EBUS-TBNA, mostly in patients with lymphoma and benign mediastinal lesions. Additionally, in 97% of EBUS-TMC samples, genetic and immunohistochemical analyses, including PD-L1 testing, were feasible, highlighting the particular value of EBUS-TMC for molecular profiling of mediastinal samples [148]. Another meta-analysis including 538 patients across 10 studies demonstrated a pooled overall diagnostic yield of 89.59% for EBUS-TMC versus 77.13% for EBUS-TBNA, with a statistically significant advantage of EBUS-TMC in lymphoma and benign disorders. The safety profile of EBUS-TMC was also confirmed. Clinically significant bleeding occurred in 1.12% and pneumothorax in 0.74% of patients, with no procedure-related mortality reported [149]. A further meta-analysis including 20 studies demonstrated significantly higher diagnostic efficacy for EBUS-TMC compared with EBUS-TBNA (RD 0.30; 95% CI: 0.17–0.44; *p* < 0.001). Regarding the complications, there was no significant differences in pneumothorax or bleeding risk between the two techniques [150].

For molecular testing in NSCLC, TMC samples have also shown advantages due to their larger size and higher tumor cellularity [151]. In a study EBUS-TMC demonstrated a significantly higher diagnostic yield compared with EBUS-TBNA (90.4% vs. 67.7%; *p* < 0.001), including in heterogeneous lesions (92.3% vs. 69.4%; *p* = 0.011). EBUS-TMC samples also showed higher tumor cellularity (65% vs. 30%; *p* < 0.001) and improved NGS success rates (85.4% vs. 61.9%; *p* = 0.035) [152]. Similarly, a study reported a higher diagnostic yield for EBUS-TMC compared with EBUS-TBNA (91.2% vs. 56.2%; *p* < 0.001) and demonstrated that immunohistochemistry was feasible more frequently with cryobiopsy (88.9%) than with needle aspiration (40.7%; *p* < 0.001) [153]. The larger tissue fragments obtained by EBUS-TMC (approximately 3.5 ± 0.7 mm) are associated with higher nucleic acid yields, with mean extracted DNA and RNA concentrations of 97.2 ± 22.4 ng/µL and 26.6 ± 4.9 ng/µL, respectively, providing adequate material for both histomorphological assessment and comprehensive molecular testing [154].

Despite these promising results, EBUS-TMC remains a relatively new technique, and large prospective head-to-head studies comparing EBUS-TMC with conventional EBUS-TBNA or EBUS-guided forceps biopsy are still limited. Therefore, additional evidence is required before widespread implementation of EBUS-TMC in routine clinical practice can be recommended [155].

## 5. Future Directions

### 5.1. Thin Convex Probe EBUS (TCP-EBUS)

A third generation of thin convex probe EBUS (TCP-EBUS) scope is now available to overcome the limitations of currently commercially available convex probe EBUS (CP-EBUS) bronchoscopes, which have limited ability to explore segmental bronchi, particularly in the upper lobes, mainly due to restricted angulation and their relatively larger diameter [156,157].

The newly developed TCP-EBUS scope (Olympus BF-UC290F) provides improved flexibility with enhanced angulation of up to 160°. It features a smaller diameter with a compact distal tip of only 6.6 mm, and the ultrasound scanning range has been increased by 5°. These improvements allow better access to segmental bronchi and smaller airways, facilitating the evaluation of peripheral lesions and lymph node staging beyond the hilar and lobar lymph nodes—areas that are often difficult to reach using standard EBUS bronchoscopes [156,157].

An initial evaluation of TCP-EBUS in a cadaveric model demonstrated greater accessibility compared with standard convex EBUS in several bronchi, with statistically significant differences (*p* < 0.05). In addition, puncture success rates for simulated lesions using TCP-EBUS were significantly higher than those achieved with conventional convex EBUS in both segmental bronchial regions (85.0% vs. 60.0%, *p* < 0.001) and subsegmental bronchial regions (84.4% vs. 38.9%, *p* < 0.001) [157].

Notably, TCP-EBUS utilizes only a thin 25-gauge needle due to the smaller working channel of the scope. However, several studies have demonstrated that samples obtained using a 25-gauge needle are comparable to those obtained with a 22-gauge needle in terms of diagnostic yield and their suitability for genetic analysis using NGS [158,159]. The adequacy of samples obtained with a 25-gauge needle using TCP-EBUS specifically for molecular analysis has not yet been systematically investigated. The suitability of these samples may also be influenced by the location of the target lesions. In addition, the integration of a 1.1 mm cryoprobe for EBUS-TMC, which can be used through the TCP-EBUS scope, may help overcome potential sampling limitations associated with the smaller needle size. Nevertheless, this approach requires further clinical evaluation.

### 5.2. Artificial Intelligence (AI) in EBUS

Recent advances in artificial intelligence (AI) and machine learning, particularly deep learning and convolutional neural networks, have significantly impacted medical imaging. The integration of these technologies into the field of interventional pulmonology has improved procedural planning and enabled real-time assessment, including intraprocedural tissue evaluation. This integration has the potential to improve diagnostic yield and enhance the quality of obtained samples for subsequent molecular and genetic analyses [160]. Several studies have investigated the reliability of AI models applied to grayscale EBUS images for differentiating metastatic from benign lymph nodes. The reported diagnostic accuracy of these models varies considerably, ranging from 73% to 97% across different trained algorithms [161,162,163].

However, these findings remain preliminary, as most studies are limited by single-center datasets, relatively small sample sizes, and restricted external validation. Furthermore, the potential utility of AI-trained models and their impact on the reliability and diagnostic accuracy of EBUS-TBNA samples for molecular analysis and NGS have not yet been adequately investigated. Multicenter studies with larger sample sizes are therefore needed to standardize the procedure and to validate the clinical applicability of AI-assisted EBUS in the diagnosis of metastatic lymph nodes, as well as to assess the adequacy of obtained samples for molecular analyses.

Additionally, the implementation of AI algorithms in ROSE is a promising approach to overcome the limitations associated with the routine use of ROSE in the bronchoscopy suite, which is often constrained by the limited availability of pathologists/cytologists. Recent studies have demonstrated that AI systems are effectively capable of analyzing ROSE smears with high accuracy of identification if cancerous cells and may thereby enhance the diagnostic efficiency and adequacy of EBUS-TBNA samples [164,165]. Beyond the recognition of tumor cellularity, recent ML models have increasingly been investigated for their ability to integrate multidimensional data sources, such as whole-transcriptome RNA sequencing and airway epithelial transcriptional profiling, thereby improving diagnostic yield [166]. Furthermore, ML models based on programmed cell death (PCD) signatures have demonstrated promising prognostic potential and may help identify patients at risk of resistance to immunotherapy, thereby contributing to improved patient stratification and personalized treatment strategies for lung adenocarcinoma [167].

However, the currently available evidence supporting the clinical validity of AI-driven models for immunohistopathological sample analysis remains limited and is largely derived from early exploratory studies. Greater data availability, improved integration with established procedural technologies, and further prospective multicenter studies are essential to validate these models and facilitate their implementation in routine clinical practice.

## Data Availability

This is a review article; no new datasets were generated or analyzed during the current study.

## Abbreviations

The following abbreviations are used in this manuscript:

ACCP	American College of Chest Physicians
ADC	Antibody–drug conjugate
AI	Artificial intelligence
ALK	Anaplastic lymphoma kinase
AMP	Association for molecular pathology
CAP	College of American Pathologists
CNS	Central nervous system
CP-EBUS	Convex probe EBUS
CtDNA	Circulating tumor DNA
CTLA-4	Cytotoxic t-lymphocyte-associated antigen 4
DC	Dendritic cells
EBUS-TBNA	Endobronchial ultrasound-guided transbronchial needle aspiration
EBUS-TMC	EBUS-Transbronchial Mediastinal Cryobiopsy
EGFR	Epidermal growth factor receptor
EpCAM	Epithelial cell adhesion molecule
FISH	Fluorescence in situ hybridization
FGFR	Fibroblast growth factor receptor
GTP	Guanosine triphosphate
HER2	Human epidermal growth factor receptor 2
IASLC	International Association for the study of Lung Cancer
ICI	Immune checkpoint inhibitor
IHC	Immunohistochemistry
KEAP1	Kelch-like ECH-associated protein 1
KRAS	Kirsten rat sarcoma viral oncogene homolog
mTOR	Mammalian target of rapamycin
MET	Mesenchymal–epithelial transition
NK	Natural killer
NGS	Next-generation sequencing
NSCLC	Non-small cell lung cancer
ORR	Objective response rates
PCR	Polymerase chain reaction
PD-1	Programmed cell death protein-1
PD-L1	Programmed death-ligand 1
PFS	Progression-free survival
PI3K	Phosphatidylinositol 3-kinase
PlGF	Placenta growth factor
RT-PCR	Reverse transcription polymerase chain reaction
ROS1	ROS proto-oncogene 1
ROSE	Rapid on-site evaluation
SG	Sacituzumab Govitecan
SqCC	Squamous cell carcinoma
STK11	Serine/Threonine Kinase 11
TCP-EBUS	Thin convex probe EBUS
TKIs	Tyrosine kinase inhibitors
TNM	Tumor–node–metastasis
TPS	Tumor proportion score
TROP-2	Trophoblast cell surface antigen 2
VEGF	Vascular endothelial growth factor
